# Exploring the Biomedical and Environmental Application of Silver Oxide Nanoparticles Derived From *Citrus sinensis* Peel: A Valorization Approach

**DOI:** 10.1002/fsn3.71818

**Published:** 2026-05-19

**Authors:** Diksha Sharma, Prabhleen Kaur, Aparajita Bhasin, Samriti Guleria, Halis Simsek, Prashant Anil Pawase

**Affiliations:** ^1^ Department of Food Science and Technology Khalsa College Amritsar India; ^2^ Department of Food Technology and Nutrition Lovely Professional University Phagwara India; ^3^ Department of Agricultural & Biological Engineering Purdue University West Lafayette Indiana USA; ^4^ MIT INSPIRE (Institute for Sponsored and Innovative Research), MIT Art Design and Technology University Loni Kalbhor India

**Keywords:** Antibacterial, Green synthesis, Photocatalysis, Silver Oxide Nanoparticles, Waste Valorization

## Abstract

The use of agricultural waste offers an eco‐friendly and sustainable way to synthesize functional nanomaterials. In this study, silver oxide nanoparticles (AgO‐NPs) were synthesized by using peel extracts from *Citrus deliciosa* (CD) and *Citrus nobilis* (CN) through a green method. The bioactive compounds present in peel extracts act as natural reducing and stabilizing agents. UV–Visible spectroscopy showed a distinctive absorption peak in the range of 400–460 nm, confirming the successful formation of AgO‐NPs. Dynamic Light Scattering analysis revealed that the AgO‐NPs had hydrodynamic diameters between 14 to 55 nm. Scanning Electron Microscopy confirmed that the nanoparticles were mostly spherical. Fourier transform infrared spectroscopy identified functional groups on the nanoparticle surface. AgO‐NPs derived from CD peel extract showed significant antibacterial properties, with inhibition zones ranging from 19 to 21 mm against *Shigella dysenteriae* , *Escherichia coli* , *Staphylococcus aureus* , and *Listeria monocytogenes* . In antioxidant tests using the DPPH free radical scavenging assay, the CD‐derived AgO‐NPs achieved 65% inhibition at a concentration of 70 μg/mL, showing their strong ability to neutralize reactive oxygen species. Additionally, the α‐amylase and α‐glucosidase inhibition assays demonstrated notable in vitro enzyme inhibition by the AgO‐NPs. Beyond their biomedical uses, the CD‐derived AgO‐NPs showed strong photocatalytic activity, achieving 73.51% degradation of methylene blue (MO) and 71% degradation of methyl orange (MO) when exposed to UV light. Together, these multifunctional properties highlight the usefulness of AgO‐NPs made from citrus waste in both medical and environmental applications. Using fruit peel as a precursor not only adds value to agricultural waste but also supports circular economy efforts by promoting eco‐friendly and sustainable development of nanomaterials.

## Graphical abstract

The graphical abstract illustrates that the AgO‐NPs were synthesized by utilizing the citrus peel extract derived from *Citrus nobilis* and *Citrus deliciosa*. The bioactive compounds in the peel extract are effective natural reducing and stabilizing agents during the nanoparticle's synthesis process. The characterization was done by using various techniques such as UV–visible spectroscopy, Dynamic light scattering, Scanning electron microscopy, Thermogrametric, and X‐ray diffraction. The UV–Vis spectroscopy showed a distinct absorption peak, while the dynamic light scattering analyzed the particle size distribution. Scanning electron microscopy further reveals the morphology and surface features of the synthesized nanoparticles. Thermogravimetry, and X‐ray diffraction provide the information related to thermal stability and crystallinity of AgO‐NPs, respectively. The findings of this study highlight the multifunctional potential of AgO‐NPs. Antibacterial activity against microbial strains, alongside notable antidiabetic potential. In addition, the nanoparticles exhibit effective catalytic performance in dye degradation, indicating their applicability in environmental remediation. Overall, this work presents an eco‐friendly, cost‐effective strategy for nanoparticle synthesis using agro‐waste, with promising implications in biomedical and environmental applications.

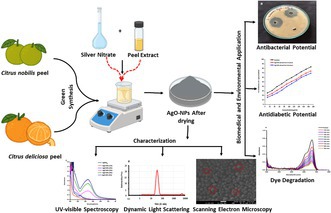

## 1 Introduction

The growing burden of environmental pollution, along with concurrent increases in microbial resistance and metabolic disorders, has challenged the world to find multifunctional materials that address the crisis on both fronts. Pollution and water contamination from industrial practices (coal, textile, and all sectors of agriculture) and uncontrolled agricultural runoff, as well as unwanted synthetic chemical contamination from the overuse of synthetic dyes and pharmaceuticals, have created an immense burden on water quality, affecting ecosystems and human health (Kumar 2023). Subsequently, the emergence of antibiotic‐resistant pathogens and the global prevalence of chronic conditions such as diabetes have called for the rapid development of new therapeutic approaches to combat diseases in the most effective and sustainable ways possible (Ramesh et al. 2024). Nanotechnology is a promising new multidisciplinary area that may simultaneously target biomedical and environmental crises (Skiba and Vorobyova 2019). The focus of this special issue is nanoparticles, particularly metal‐based nanoparticles, including silver oxide nanoparticles (AgO‐NPs), recognized for their potent antibacterial, antioxidant, and catalytic properties (Siddiqui et al. 2023; Siddiqui 2023). The ability of these scaling nanoparticles to generate reactive oxygen species (ROS), disrupt microbial membranes, and chemically couple together in redox reactions makes them suitable for both environmental and biomedical uses (Chandraker et al. 2020). Moreover, for the fabrication of nanoparticles, conventional chemical and physical fabrication methods are involved, such as chemical reduction, sol–gel processing, thermal decomposition, laser ablation, and sputtering, which are widely employed due to their ability to produce materials with controlled size and morphology (Gao et al. 2022; Faisal et al. 2021; Faisal et al. 2022). However, these approaches often involve the use of hazardous reducing agents, organic solvents, stabilizers, or require high temperatures, vacuum conditions, and sophisticated instrumentation (Bhat et al. 2019; Daghestani et al. 2020). Such requirements increase energy consumption, operational cost, and environmental burden, while also raising concerns regarding residual toxicity and biocompatibility (Nirmal et al. 2023). In addition, the generation of chemical waste and the need for post‐synthesis purification steps can limit scalability and sustainability (Kumar and Pandey 2017; Almukaynizi et al. 2022). As a result of all of this, interest is shifting to green synthesis ways of using biological resources to generate nanoparticles in a safer “greener” manner (Kumar 2023). Green synthesis can facilitate the control of particle shape and dispersion, making it a finer approach to investigating the activity of the nanoparticles. In addition, when using raw material, plant‐based synthesis processes are inexpensive and well‐suited to large‐scale production methods when utilizing agricultural or food processing wastes(Kaur et al. 2023). Therefore, by allowing for the dual‐processing of resources while fabricating nanomaterials, green synthesis approaches can ultimately be more desirable in ways of sourcing for environmental and biomedical nanotechnology. By using plant resources, specifically agro‐waste resources, researchers developed multifunctional nanoparticles while allowing for waste reduction and proper resourcing (Kandemir et al. 2022; Dell'AnnunziataDell'Annunziata et al. 2024). This supports environmentally responsible and circular economy practices. Orange (*Citrus sinensis*), one of the most widely produced fruits around the globe, is recognized for its sweet, juicy, aromatic, and mildly acidic pulp, along with its well‐documented medicinal and therapeutic benefits (Eze et al. 2022; Ahmed et al. 2023). The orange juice processing industry generates a huge amount of waste each year, particularly peels, which are often discarded (Kumar et al. 2023; Kumar et al. 2024), resulting in undesirable waste and environmental pollution. As a result, there is an increasing interest in using orange peel waste in order to extract useful bioactive compounds by simple and easy methods (Wang et al. 2022; Ahmed et al. 2023). It has been established that orange peels possess a rich, diverse collection of phytochemicals, meaning that this waste may allow for an extensive range of biological activities such as antioxidant, antidiabetic, antimicrobial, and anti‐inflammatory activities (Hassan et al. 2021; Saini et al. 2022; Ahmed et al. 2023). Of note, its antioxidant capability is of prime significance due to the high content of primary and secondary metabolites such as proteins, carbohydrates, phenolic compounds, flavonoids, carotenoids, essential oils, and ascorbic acid (Mani et al. 2021; Singh 2023). Flavonoids, in particular, the most numerous class of phenolic compounds, are also of considerable importance, and it is worth noting that anthocyanidins have very powerful reducing and antioxidant abilities (Chinnathambi et al. 2021; Zannotti et al. 2024). Carotenoids like α‐carotene, β‐carotene, lycopene, and xanthophylls also possess bioactivity. Ascorbic acid (vitamin C) is a second significant phytochemical that has been shown to act as a powerful antioxidant and reductant (Viñas‐Ospino et al. 2023; Kumar et al. 2024). Due to the wealth of phytochemicals present, orange peel has been used successfully as a biological template in the green synthesis of metal and metal oxide nanoparticles, which provide a green route to producing nanomaterials for biomedical and environmental purposes (Chidambara Murthy et al. 2022). This study describes a green synthesis method from orange peel waste, variable, and many agricultural by‐products, to synthesize silver oxide nanoparticles (AgO‐NPs). The nanoparticles were characterized and tested for functional properties such as Antibacterial (Almukaynizi et al. 2022; Bhat, Al‐Dbass, et al. 2024; Bhat, Alonazi, et al. 2024), antioxidant and antidiabetic, and degradation of synthetic dyes, suggesting treatment options for wastewater. A single nanoparticle system with such a range of biological activity is rare. The nature of the green synthesis means the AgO‐NPs are viewed from a dual biomedical and environmental perspective with a single investigation. This holistic study highlights many aspects of citrus peel waste and encourages a circular economy and development goals by contributing to the green production of nanomaterials and developing new techniques for environmental remediation. Overall, the research provides a significant and valuable contribution to the nascent area of green nanotechnology and waste valorization.

## 2 Materials and Methods

Silver nitrate, sodium hydroxide, hydrochloric acid, and 2,2‐diphenyl‐1‐picrylhydrazyl (DPPH) were obtained from Sigma‐Aldrich (Merck, Germany). Methylene blue and methyl orange were obtained from LOBA Chemie (Mumbai, India). Two gram‐positive pathogens, *Staphylococcus aureus* (MTCC 3160), *Listeria monocytogenes* (MTCC 839), and two gram‐negative strains, *Escherichia coli* (MTCC 1302), *Shigella dysenteriae* (MTCC 5151), were acquired from the Microbial Type Culture Collection (MTCC), Institute of Microbial Technology (IMTECH), Chandigarh, India. Deionized water was used to prepare all chemicals solutions.

### 2.1 Collection and Preparation of the Sample

Peel samples of *Citrus deliosa* (CD) and *Citrus nobilis* (CN) were taken from the canteen of Khalsa College, Amritsar, and the nearby Juice bar of Gurdaspur, respectively. The peels collected were cleaned and oven‐dried at 70°C. Following drying, the samples were ground into powder in a laboratory grinder and stored in the dark under airtight conditions. Proximate composition analysis was conducted to determine moisture, ash, crude protein, crude fat, and total carbohydrate. The AOAC methods were utilized to perform proximate analysis. The carbohydrate content was calculated by determining the differences. The total phenolic content (TPC) and total flavonoid content (TFC) of powders from CD and CN peels were determined using a method by Hussain, Fareed, et al. (2022), and Hussain, Qureshi, et al. (2022):

(1) $$\text{Total carbohydrate}=100-\left(\mathrm{ash}\;\text{content}+\text{moisture content}+\mathrm{fat}+\text{protein}\right)$$

### 2.2 Preparation of Orange Peel Extract

For the preparation of the extract, a modified protocol based on Hussain, Fareed, et al. (2022), Hussain, Qureshi, et al. (2022), and Hussain, Rahim, et al. (2022) was followed. In brief, 5.0 g of the powdered sample was mixed with 100 mL of double‐distilled water and heated in a water bath at 70°C for 35 min under gentle conditions. After heating, the extract was allowed to cool to room temperature and then centrifuged at 10,000 × g for 20 min to increase the extraction capacity. The supernatant was removed and filtered using Whatman No. 1 filter paper, and the clear filtrate was stored at 4°C for subsequent analysis. The aqueous extract was also characterized using Fourier Transform Infrared (FTIR) spectroscopy to identify possible functional groups.

### 2.3 Fabrication of Silver Oxide Nanoparticles (AgO‐NPs)

The modified version of the method was utilized for the fabrication of AgO‐NPs (Guleria et al. 2025). Initially, a 10 mM silver nitrate (Ag‐NO_3_) solution was prepared, and the pH was adjusted to 8 by using 0.1 N NaOH and HCL. Then, 25 mL of metal solution was taken from the stock solution and placed on a magnetic stirrer. Then, citrus peel extract was added dropwise into the AgNO_3_ solution under constant stirring (500 rpm). The mixture was held at 60°C for 20 min to allow the AgO‐NPs to form. A visible color change was evidenced in the successful fabrication of AgO‐NPs. In order to find the optimal stabilization of AgO‐NPs, multiple concentrations of peel extracts were tested. For *Citrus deliosa* (CD) peel extract, concentrations of the peel extract were 0.5%, 1%, 1.5%, 2%, and 2.5% were used. For *Citrus nobilis* (CN) peel extract, the extracts were tested at concentrations of 1%, 2%, 3%, 4%, and 5%.

### 2.4 Characterization of AgO‐NPs

A spectrophotometric and analytical technique was applied for the characterization of AgO‐NPs synthesized through the orange peel extract. The optical properties and localized surface plasmon resonance (LSPR) characterizations were examined using UV–Vis spectroscopy. Dynamic Light Scattering (DLS) was used to determine the particle size distribution. The surface functional groups were investigated using Fourier Transform Infrared Spectroscopy (FTIR). Therefore, the morphologies of the nanoparticles were visualized through Scanning Electron Microscopy (SEM), while Elemental Composition was evaluated using Energy Dispersive X‐ray Spectroscopy (EDS). The thermal stability of the nanoparticles was reported using Differential Scanning Calorimetry (DSC), while the decomposition behavior was characterized using Thermogravimetric Analysis (TGA). The crystallographic structure, phase transitions, and melt behavior of the AgO‐NPs were determined by X‐ray Diffraction (XRD) analysis.

### 2.5 Application of AgO‐NPs

Fabricated AgO‐NPs were evaluated for their biomedical and environmental potential.

#### 2.5.1 Biomedical Potential of AgO‐NPs

Synthesized AgO‐NPs were evaluated for their antibacterial, antioxidant, and antidiabetic potential.

#### 2.5.2 Assessment of Antibacterial Potential of AgO‐NPs

The antibacterial efficacy of AgO‐NPs was determined by the agar well diffusion method according to Guleria, Chawla, et al. (2024). Two Gram‐positive *S. aureus* , *L. monocytogenes*, and two Gram‐negative *S. dysenteriae*, *E. coli*. bacterial strain was studied. Mueller‐Hinton agar plates were prepared with nutrients, and wells were removed. Then, 10 μL of AgO‐NPs mixed with dimethyl sulfoxide (DMSO) was added to the wells. Chloramphenicol was used as a positive control, and DMSO as a negative control. The inoculated plates were incubated at 37°C for 24 h. After 24 h, the zones of inhibition were measured in millimeters. Reliable results were demonstrated because a triplicate of each of the experiments was completed. The minimum inhibitory concentration (MIC) and minimum bactericidal concentration (MBC) were determined using the broth dilution method (Relhan et al. 2025).

#### 2.5.3 Antioxidant Activity by DPPH Scavenging Assay

The radical scavenging assay was performed based on the methodology by Guleria, Simsek, et al. (2024) to ascertain the antioxidant activity of green‐synthesized AgO‐NPs. To each of the test tubes, 3 mL of the DPPH solution was added to 1 mL of the different concentrations of nanoparticles. The mixtures were incubated in the dark at room temperature for 30 min. After incubation, the absorbance of each sample was measured at 517 nm using a UV–Vis spectrophotometer, against a blank containing only the solvent without DPPH. The percentage of radical scavenging activity (inhibition) was calculated using Equation (2).

(2) $$\%\text{inhibition}=\frac{A_{\text{control}}-A_{\text{test}}}{A_{\text{test}}}\times 100$$

where $A_{\text{control}}$ is the absorbance of the control reaction, and $A_{\text{test}}$ is the absorbance in the presence of AgO‐ NPs.

#### 2.5.4 Antidiabetic Assay

The anti‐diabetic potential of AgO‐NPs was evaluated using the α‐amylase and α‐glucosidase inhibition assay reported by Majumder et al. (2025).

##### 2.5.4.1 α‐Amylase Inhibition Assay

The amylase inhibition activity was evaluated using the procedure described by Panda et al. (2024) with a slight change. Different concentration of AgO‐NPs was prepared in DMSO. The synthesized AgO‐NPs (20 μL) of different concentrations were added to the test well, followed by the addition of 25 μL of α‐amylase solution (50 μg/mL), phosphate buffer (15 μL) of α‐amylase, and starch (4 μL). Then the mixture was incubated at 50°C for 30 min. Next, 20 μL of 1 M HCl and 90 μL of iodine solution were loaded into each well. The mixture was then subjected to centrifugation at 3000 rpm for 5 min at 4°C, from which the supernatant was collected for the optical density measurement at 540 nm in a spectrophotometer. In addition, acarbose was used as a positive control, which is known as an α‐amylase inhibitor. Next, 20 μL of 1 M HCl and 90 μL of iodine solution were loaded into each well. The experiments were repeated three times for each concentration to ensure accuracy. Amylase inhibitory activity was calculated by Equation (3).

(3) $$\text{Amylase inhibition}\;\left(\%\right)=\frac{{\text{sample}}_{\mathrm{Abs}}-{\text{Negative control}}_{\mathrm{Abs}}}{{\text{Blank}}_{\mathrm{Abs}}-{\text{Negattive control}}_{\mathrm{Abs}}}\times 100$$

where Abs represents the absorbance, and the concentration required to inhibit 50% of α‐amylase (IC_50_) was determined for acarbose.

##### 2.5.4.2 α‐Glucosidase Inhibition Assay

A glucosidase inhibition assay was conducted to evaluate the α‐glucosidase inhibitory capability of AgO‐NPs using the protocol adapted from (Guleria, Simsek, et al. 2024). To activate α‐glucosidase, 50 mL of phosphate buffer (pH 6.8), along with 100 mg of bovine serum albumin, was mixed at the same time. After 5 min, 490 μL of phosphate buffer (pH 6.8), along with 250 μL of p‐nitrophenyl‐d‐glucopyranoside (5 mM), was mixed and incubated at 37°C for 15 min. Following incubation, ZnO‐NPs were mixed with 250 μL of α‐glucosidase and incubated for 15 min at 37°C. The reaction was terminated with 2 mL of Na_2_CO_3_ (200 mM) solution and measured at 400 nm. The experiment was executed in triplicate to provide accuracy, with acarbose being a positive control. Glucosidase inhibition was determined using Equation (3) for amylase inhibitory activity.

#### 2.5.5 Environmental Potential of AgO‐NPs

The photocatalytic dye degradation potential of AgO‐NPs was studied. In order to study the removal of dye by AgO‐NPs, methylene blue (MB), and methyl orange (MO) dyes were selected as the model pollutants. The method used and experimental procedures were slightly modified from Lotfi et al. (2024). In the first instance, 10 mM solutions of both dyes were made. The AgO‐NPs were added to the dye solutions and stirred in the dark with a magnetic stirrer for 10 min at 150 rpm to achieve adsorption/desorption equilibrium. After 10 min, 30 mL of dye solution was removed to a conical flask and placed on a magnetic stirrer, and 2 mL of AgO‐NPs suspension was added. Then, the mixture was irradiated with ultraviolet (UV) light. At predetermined time intervals, 3 mL aliquots were withdrawn and centrifuged to remove residual nanoparticles. The clear supernatant was collected, and the remaining concentration of dye was determined by measuring absorbance with a UV–Vis spectrophotometer. The progressive decline in dye absorbance was used to quantify the photocatalytic activity of AgO‐NPs under UV exposure, assessing their effectiveness in degrading MB and MO by using Equation (4).

(4) $$\mathrm{Dye}\;\text{degradation efficiency}=\frac{C_{0}-C_{t}}{C_{0}}\times 100$$

where $C_{0}$ and $C_{t}$ were the initial and remaining dye concentration (mg/L) of the aqueous solution at time 0 and *t*.

## 3 Results and Discussion

### 3.1 Proximate Analysis of Collected Samples

The proximate composition analysis of the citrus peel powders revealed notable nutritional value. CD peel exhibited a moisture content of 8.5%, while CN showed a slightly higher value at 9.2%. Ash content was more pronounced in CD (6.43%) compared to CN (5.72%), suggesting a greater concentration of essential inorganic minerals in CD. The protein content was also superior in CD (9.02%) relative to CN (7.85%). Both peel samples had minimal fat content, recorded at 2.15% for CD and 1.78% for CN. In contrast, CN demonstrated a slightly higher carbohydrate content (73.56%) than CD (71.45%). These findings are consistent with previously published literature, where citrus peel proximate values were reported within similar ranges. According to Sogi et al. (2015a) and Sogi et al. (2015b), moisture content in citrus peels was on average 6.35%–9.82% values reported by Kumar et al. (2017), Makanjuola, Enujiugha, and Abiodun (2020), and Makanjuola, Enujiugha, and Omoba (2020) ash values were within the range of 4.12%–7.89% ash, reflecting the changing mineral compositions. Crude protein levels highlighted by Hussain, Fareed, et al. (2022), Hussain, Qureshi, et al. (2022), and Hussain, Rahim, et al. (2022) were likely ranging between 5.42% and 10.65% confirming that citrus peels are a moderate protein source. Fat was consistently low across all studies at 1.20%–3.10%. Total carbohydrates were at a relatively high proportion, and often fall within the range of 68.5%–78.6%, which highlights the high carbohydrate composition in citrus peels.

Additionally, TPC analysis revealed values of 52.6 mg GAE/g dry weight for CD and 48.9 mg GAE/g dry weight for CN, indicating a strong antioxidant potential in both samples. In parallel, TFC was measured at 23.6 mg QE/g dry weight for CD and 18.6 mg QE/g dry weight for CN, highlighting the presence of bioactive compounds with potential health‐promoting properties.

### 3.2 Mechanism for the Fabrication of AgO‐NPs

The combination of phytochemicals, the pH of the reaction solution, and dissolved O_2_ in the solvent drives the synthesis of AgO‐NPs from citrus peel extracts, as represented in Figure S1. Citrus peel extracts contain numerous phenolic compounds, flavonoids, organic acids, and proteins. These substances contain functional groups such as hydroxyl (‐OH), carbonyl (> C=O), and carboxyl (‐COOH), which can chelate silver ions or participate in the reduction of Ag^+^ to metallic silver (Ag^0^) (Iravani 2011). At the experimental conditions of pH 8 and atmospheric conditions (ambient), dissolved oxygen facilitates partial oxidation processes (Mittal et al. 2013). The reduction of silver to metallic Ag^0^ does not occur, but instead the coordinated silver becomes oxidized and forms Ag‐O bonds, resulting in the formation of AgO‐NPs through nucleation processes (Raut et al. 2010). Phenolic and flavonoid compounds act as both reducing and stabilizing agents, allowing for the formation of AgO (silver oxide) rather than Ag (metallic silver). Their controlled strength of reducing favors the formation of AgO‐NPs. Proteins and polysaccharides further stabilize the growing nanoparticles through surface adsorption, preventing aggregation and maintaining the oxide phase (Ahmed et al. 2016; Singh and Yadav 2021).

**TABLE 1:** The particle size of silver oxide nanoparticles (AgO‐NPs) derived from different concentrations of *Citrus deliosa* (CD) and *Citrus nobilis* (CN) onion peel extracts.

Orange peel extracts	Concentration of extract used (%)	Particle size of AgO‐NPs (nm)
*Citrus nobilis* (CN)	0.5	18.12 ± 2.45^c^
	1.0	52.96 ± 3.25^b^
	1.5	38.28 ± 4.52^a^
	2.0	15.11 ± 1.15^d^
	2.5	29.45 ± 5.55^e^
*Citrus deliosa* (CD)	1	47.15 ± 1.11^d^
	2	61.61 ± 2.52^e^
	3	18.89 ± 3.07^b^
	4	14.59 ± 3.44^a^
	5	21.54 ± 4.36^c^

*Note:* These superscripts indicate whether there is a statistically significant difference between the groups.

### 3.3 UV–Visible Spectroscopy of Ago‐NPs

**FIGURE 1:**
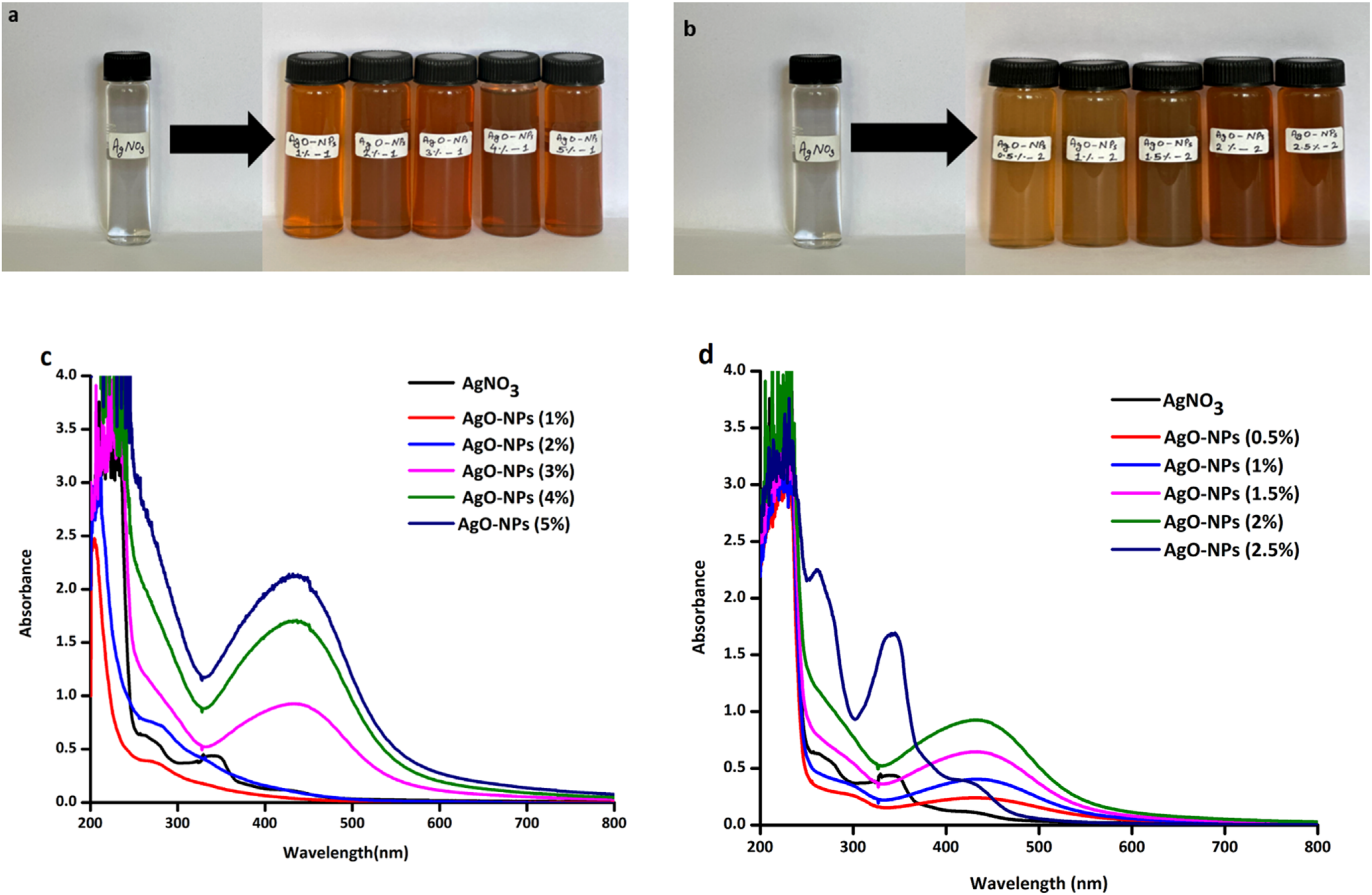
Visual confirmation of AgO‐NPs synthesized using (a) *Citrus nobilis* (CN) and (b) *Citrus deliosa* (CD) extract, (c) UV–visible spectroscopy of CN‐derived AgO‐NPs, and (d) CD‐derived AgO‐NPs.

The initial indication of AgO‐NPs synthesis was evidenced by a noticeable shift in solution color from light brown to deep brown (Figure 1a,b), signifying the reduction of silver ions. This color transformation arises from the phytochemicals present in the orange peel extract, which function as reducing and capping agents. These phytoconstituents actively reduce Ag^+^ ions to elemental silver (Ag^0^), thereby initiating nucleation and subsequent nanoparticle formation (Siddiqui et al. 2023; Siddiqui 2023; Khan et al. 2024). To validate the nanoparticle synthesis, UV–Visible spectroscopic analysis was conducted. In the measured spectra (Figure 1c,d), we see a distinct absorption peak at approximately 400–480 nm, indicating observable AgO‐NPs. That distinctly recognizable peak is attributed to the surface plasmon resonance (SPR) effect. It occurs from a collective oscillation of conduction electrons on the nanoparticle surface when exposed to specific frequencies of light, leading to enhanced optical absorption at a specific wavelength (Singh et al. 2021; Sahu et al. 2022; Abdullah et al. 2023).

### 3.4 Characterization of Silver Oxide Nanoparticles

The synthesized nanoparticles were characterized for their size, shape, functional groups, and thermal stability and cystanility.

#### 3.4.1 Particle Size and Zeta Potential

**FIGURE 2:**
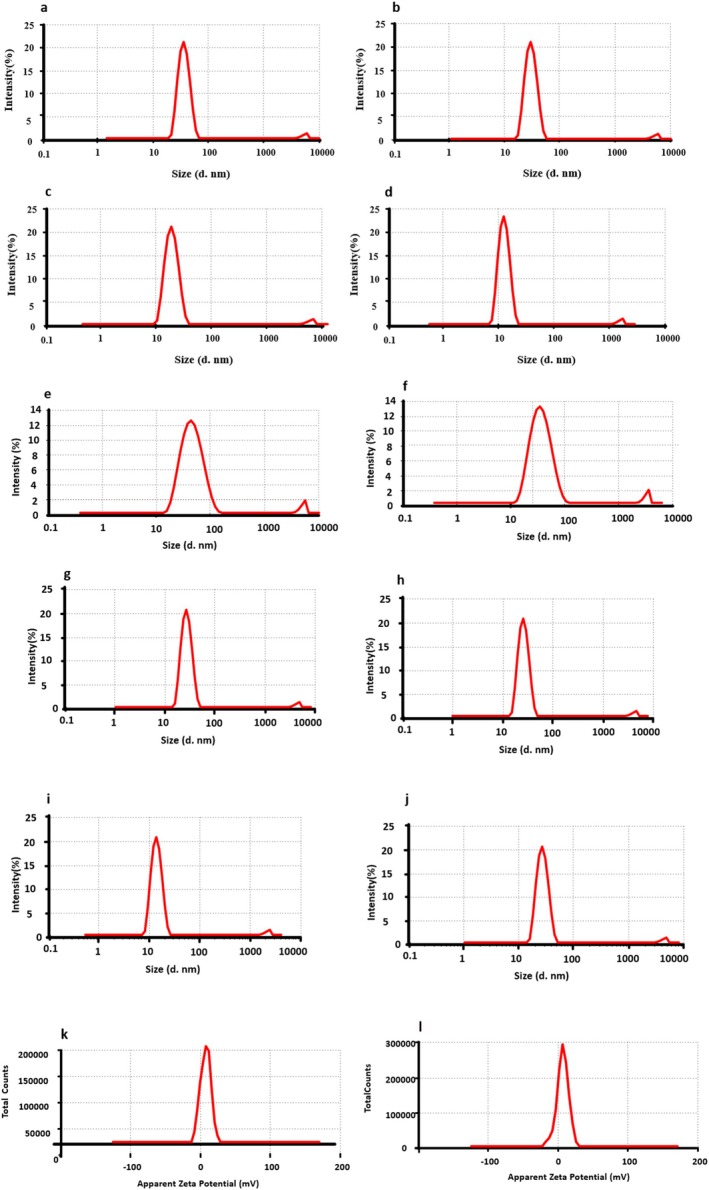
The average particle size of AgO‐NPs synthesized by using CN extract of (a) 1%, (b) 2%, (c) 3%, (d) 4%, (e) 5%; CD extract (f) 0.5%, (g) 1%, (h) 1.5%, (i) 2%, (j) 2.5%; The zeta potential of AgO‐NPs with the least particle size was synthesized using (k) 4% of CN, and (l) 2% of CD extract.

Dynamic Light Scattering (DLS) analysis was performed to assess the hydrodynamic diameter of AgO‐NPs synthesized using CD and CN peel extracts. The particle size distribution of AgO‐NPs synthesized from CD ranged between 14 and 55 nm, whereas those synthesized from CN extract exhibited sizes ranging from 14 to 50 nm, as illustrated in Figure 2a–j and Table 1. The smallest average particle size for CD‐derived AgO‐NPs was recorded at 14.59 ± 3.44 nm at a 4% concentration, while the smallest size for CN‐based nanoparticles was 15.11 ± 1.15 nm observed at 2% concentration. Based on these optimal size distributions, the corresponding formulations were selected for further evaluation of surface charge stability through zeta potential measurements (Figure 2k,l). Zeta potential values in the range of ±10–±30 mV are indicative of moderate to good colloidal stability due to sufficient electrostatic repulsion among particles, whereas values near zero imply reduced repulsive forces and a higher risk of nanoparticle aggregation (Fouda et al. 2023; Bhat, Al‐Dbass, et al. 2024; Bhat, Alonazi, et al. 2024).

#### 3.4.2 Fourier Transform Infrared Spectroscopy (FTIR)

**FIGURE 3:**
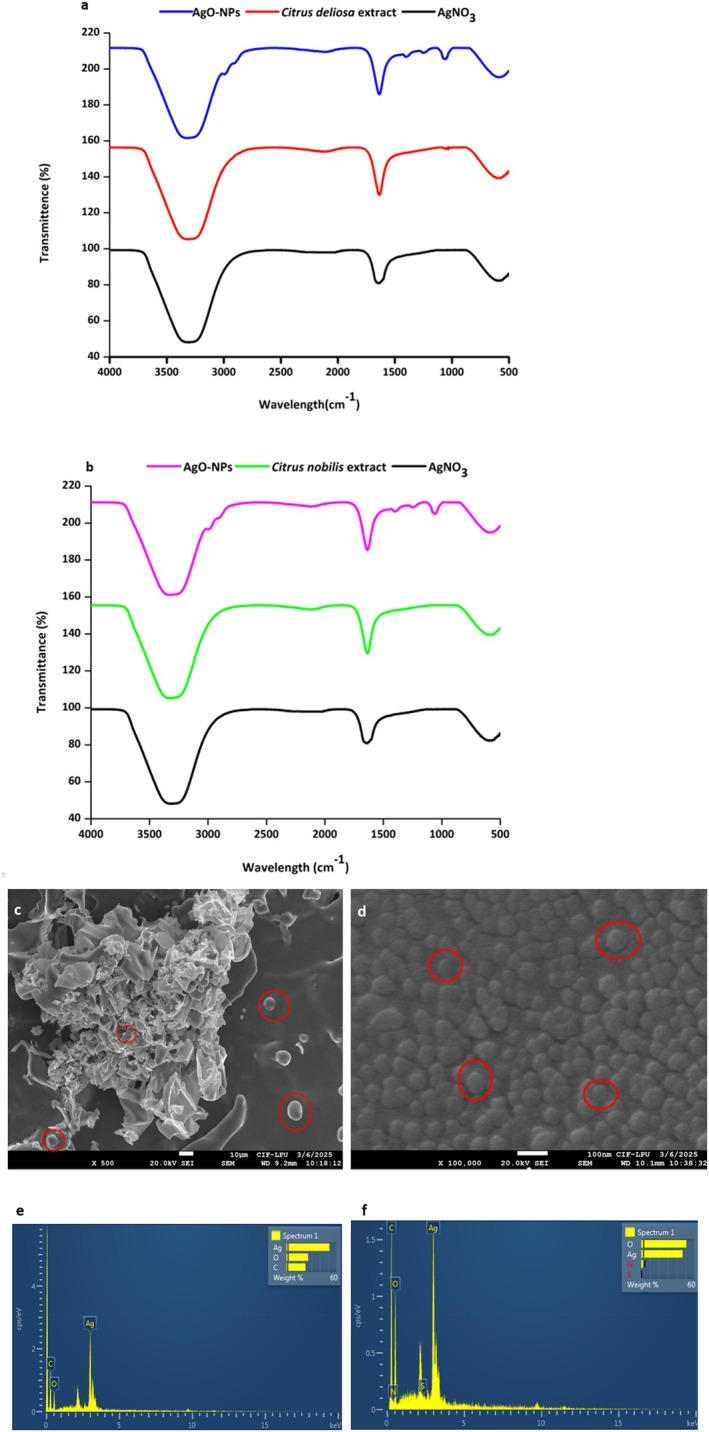
Analysis of functional groups in AgO‐NPs synthesized using (a) CN; (b) CD peel extract; (c) Surface morphology analysis of AgO‐NPs synthesized using CN; (d) CD peel extract, and (e) Elemental analysis of AgO‐NPs derived from CN; (f) CD peel extract.

The FTIR spectra of silver nitrate, CD, CN peel extract and their derived AgO‐NPs are represented in Figure 3a,b. The FTIR spectra of Silver Nitrate have a characteristic absorption band, attributed to the Nitrate ion. A very intense band located between 1380 and 1415 cm^−1^ corresponds to an asymmetric stretching mode associated with the NO_3_ confirm the presence of nitrate anions in the precursor salt. Several other Bands exist between 820 and 840 cm^−1^, which correspond to bending modes associated with nitrate ions. There are also weak bands detected between 3300–3400 cm and 1630–1650 cm. This represents the O‐H Stretching mode and the H‐O‐H bending modes of adsorbed moisture and is very typical of hygroscopic inorganic salts. Moreover, in Figure 3a, CD extract has O–H and N–H stretching bands around 3360–3390 cm^−1^ range, which correspond to phenolic, flavonoids, and protein compounds. In the spectrum of the AgO‐NPs, this band is slightly shifted, indicating that functional groups play a role in the reduction of Ag^+^ and the stabilization of AgO‐NPs (Table S1). The absorption band detected near 2920–2930 cm^−1^, assigned to aliphatic C–H stretching that corresponds to lipid moieties, is present in both the extract and the AgO‐NPs. Additionally, the spectrum also contained a strong band at 1630–1650 cm^−1^, associated with C=O stretches of amides or aromatic rings found in the extract; in the AgO‐NPs, the shift of this band indicates possible coordination of the amide or aromatic ring C=O to the silver oxide surface. Furthermore, the peaks at 1440–1455 cm^−1^ represent the C–H bending and symmetric stretching of carboxylate groups, indicating the presence of carbohydrate‐ and protein‐derived biomolecules in the extract. Additionally, absorption peaks observed in the 1240–1260 cm^−1^ and 1063–1069 cm^−1^ regions correspond to C–O and C–O–C stretching vibrations, indicating the involvement of polysaccharides and other cell wall components in nanoparticle stabilization. The low‐frequency bands found between 700 and 536–618 cm^−1^ are indicative of the Ag–O stretching vibrations that provide clear evidence of AgO‐NPs formation. The lack of nitrate‐related bands in the spectra of AgO‐NPs provides additional support for the complete reduction of Ag^+^ in the biosynthesis of NPs.

In addition, Figure 3b represents the FT‐IR spectra of AgNO_3_, CN peel extract, and CN‐derived, which reveal characteristic absorption bands associated with biomolecular functional groups involved in NPs synthesis. Figure 3b, the CN extract has a broad peak at O–H and N–H stretch around 3300–3450 cm^−1^, corresponding to phenolic, flavonoids, and protein compounds. In the AgO‐NPs spectrum, this band shows a slight shift and reduced intensity, indicating the participation of these groups in the reduction of Ag^+^ ions and stabilization of the NPs (Table S2). Furthermore, the band observed at 2920–2850 cm^−1^, corresponding to C–H stretch representing the aliphatic parts of the CN extract and AgO‐NPs, suggesting that lipids are involved in the capping and stabilization of the NPs surface. Although the C=O stretch around 1630–1650 cm^−1^ shared by both the CN extract and AgO‐NPs suggests the aggregation of carbonyl or amide group was attached to the AgO. Additional peaks at 1440–1455 cm^−1^ are attributed to C–H bending and symmetric stretching of carboxylate groups, while bands in the 1240–1260 cm^−1^ and 1063–1069 cm^−1^ regions correspond to C–O and C–O–C stretching vibrations of polysaccharides and other cell wall components. The FT‐IR spectrum of AgNO_3_ shows a strong nitrate band at 1380–1415 cm^−1^, which disappears completely in the AgO‐NPs spectrum, confirming the reduction of Ag^+^ ions. The appearance of low‐frequency bands in the 536–618 cm^−1^ region in the AgO‐NPs spectrum is assigned to Ag–O stretching vibrations, confirming the formation of AgO‐NPs. The results of the present study are consistent with the findings of Ahmed et al. (2016), Alkhulaifi et al. (2020), Jaast and Grewal (2021), Singh Mustapha et al. (2023), and Balaji et al. (2025), who reported similar observations in their research. Overall, the FTIR analysis for both CD and CN extract‐derived AgO‐NPs shows the typical involvement of plant‐derived functional groups in the reduction of silver ions and the stabilization of the resulting nanoparticles. The disappearance of the nitrate bands and the appearance of the Ag–O stretch confirm the successful formation of silver oxide, while the persistent bands from the extracts suggest that plant phytochemicals play a crucial role in nanoparticle synthesis and stabilization.

#### 3.4.3 Scanning Electron Microscopy and Elemental Analysis (SEM + EDS) of AgO‐NPs

The SEM images (Figure 3c,d) show clear differences in the shapes of AgO‐NPs made with CN and CD extracts. In the CN‐based sample (Figure 3c), the NPs are unevenly spread out, with noticeable clusters, while some distinct spherical particles are visible. These observations indicate that phytochemicals within the extract stabilized the particles to some degree, hence the irregular shape. AgO‐NPs from CD extract (Figure 3d) show more evenly distributed particles that are spherical and appear less agglomerated with a closer surface packing, suggesting that CD extract was a more effective reducing and capping agent for the synthesis of small, stable, and uniform nanoparticles. These differences further highlight the importance of the metabolites in extracts affecting a nanoparticle's shape, growth, and morphology overall. Also, the elemental composition of AgO‐NPs was assessed via Energy Dispersive X‐ray Spectroscopy (EDS) for both samples, which is provided in Figure 3e,f. The EDS spectra of AgO‐NPs based on CD and CN extract indicate important information on elemental composition and the synthesis process. The presence of a strong peak for silver in the spectra confirms the presence of silver in the nanoparticles. Additionally, the spectra for AgO‐NPs exhibit a clear peak for oxygen (O), which indicates the formation of silver oxide (AgO). The presence of peaks for carbon (C) and nitrogen (N) also occurs with silver and oxygen; therefore, it suggests compounds associated with both the citrus peel and curcuma extracts in the green synthesis process.

#### 3.4.4 Differential Scanning Calorimetry (DSC)

**FIGURE 4:**
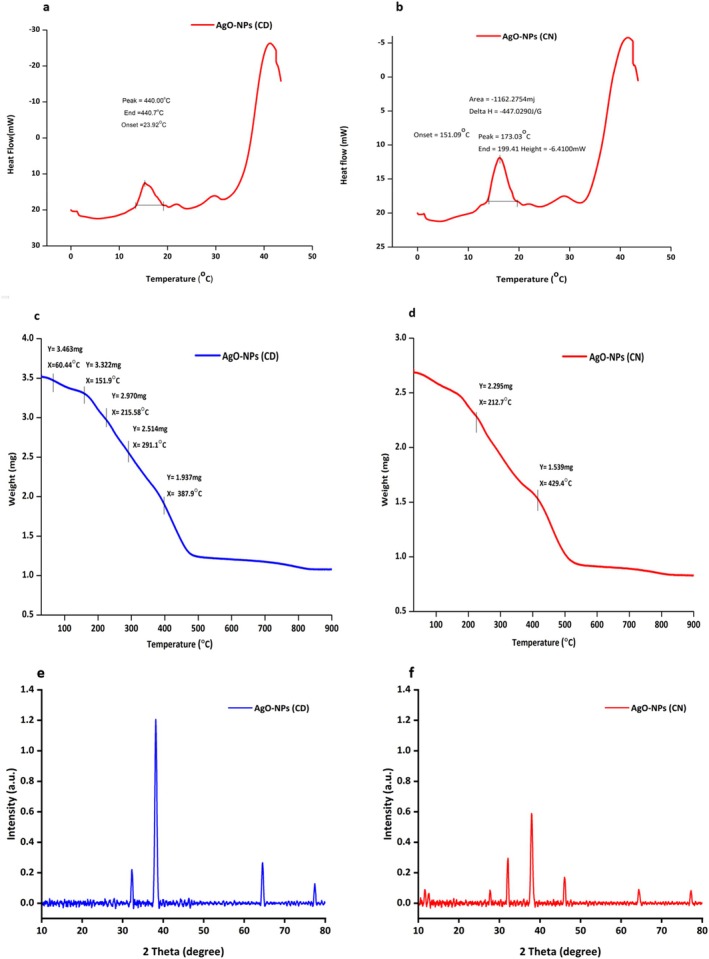
DSC analysis AgO‐NPs synthesized using (a) CD, (b) CN peel extract; (c) TGA analysis of AgO‐NPs synthesized using CD; (d) CN peel extract, and (e) XRD analysis of AgO‐NPs derived from CD; (f) CN peel extract.

The DSC profiles offer insights into the thermal behavior and stability of AgO‐NPs synthesized from CD and CN extracts (Figure 4a,b). In Figure 4a, the thermogram of AgO‐NPs from CD shows a major endothermic peak at about 440°C, starting near 23°C. This broad and sharp peak indicates strong thermal stability and suggests that the nanoparticles are highly crystalline. The higher decomposition temperature suggests that the bioactive compounds in the CD extract served as effective stabilizers. This allows the nanoparticles to handle high thermal conditions. Moreover, Figure 4b presents the DSC graph of CN‐derived AgO‐NPs, showing a significant difference in thermal response. The transition begins at 151°C, followed by a clear peak around 173°C and an endset at about 199°C. In contrast to the CD‐derived nanoparticles, this lower transition temperature shows relatively weaker thermal stability. The sharper but earlier peak indicates that the nanoparticles made from CN extract have less thermally resistant organic capping agents, which leads to earlier decomposition.

#### 3.4.5 Thermogravimetric Analysis (TGA)

The TGA spectra of AgO‐NPs from CD and CN peel extracts are shown in Figure 4c,d. For CD‐derived AgO‐NPs (Figure 4c), three distinct stages of weight loss are evident. The first loss, around 60.4°C, is due to moisture and loosely bound water evaporating from the surface of the nanoparticles. The next weight loss, near 215.6°C, likely relates to the breakdown of low‐molecular‐weight phytochemicals, such as flavonoids and phenolics. These compounds act as reducing and stabilizing agents during nanoparticle formation. A more significant degradation phase occurs between 291.1°C and 387.9°C, indicating the breakdown of more stable organic components. This is followed by the formation of thermally stable silver oxide residues. In contrast, the TGA curve for CN‐derived AgO‐NPs (Figure 4d) shows two main degradation events. The first happens at about 212.7°C and is linked to the loss of physically adsorbed bio‐organic compounds. The second event occurs around 429.4°C and suggests the decomposition of more resilient phytochemical structures or stronger interactions between the nanoparticles and the organic matrix. Above 450°C, the weight stays nearly constant, indicating the thermal stability of the remaining inorganic AgO content. When compared, the CN‐derived nanoparticles show greater thermal resistance than those from CD. This difference likely stems from variations in the amount or binding strength of phytochemicals involved during synthesis. Overall, the TGA results confirm the successful formation of thermally stable AgO‐NPs from both citrus varieties. The thermal behavior also supports the potential applicability of these nanoparticles in environments requiring thermal endurance, such as photocatalysis or material coatings. These findings align with earlier reports highlighting the role of plant‐derived capping agents in influencing the decomposition profile of green‐synthesized nanoparticles (Khan et al. 2021; Sharma 2024a, 2024b; Kalaivani and Mathubala 2024; Talib et al. 2024).

#### 3.4.6 X‐Ray Diffraction (XRD)

The diffractograms of AgO‐NPs made from CD and CN extracts show clear differences in their crystallinity and phase composition. For the AgO‐NPs from the CD extract (Figure 4e), the XRD pattern displays sharp, strong peaks at about 38°, 44°, 64°–65°, and 77°–78°, which align with the (111), (200), (220), and (311) planes of face‐centered cubic (fcc) silver (Ag). This indicates high crystallinity and well‐formed nanoparticles. Additionally, peaks related to Ag_2_O appear at around 32°–33° and approximately 54°, indicating the presence of silver oxide along with metallic silver. The narrow peaks suggest that the nanoparticles are larger and have fewer defects, which contributes to their improved stability. In addition, the CN‐derived AgO‐NPs (Figure 4f) show broader, less intense peaks. This indicates smaller crystallites or greater lattice strain, resulting in a lower degree of crystallinity than the CD‐derived nanoparticles. While the Ag_2_O peaks are still visible, they are less prominent, suggesting a smaller amount of silver oxide in the CN‐based nanoparticles. These differences in crystallinity and intensity imply that the CD extract promotes the formation of larger, more crystalline Ag/Ag_2_O nanoparticles. In contrast, the CN extract leads to smaller, more amorphous particles. This variation in structural traits can affect the nanoparticles' reactivity, stability, and potential uses, especially in fields like antibacterial treatments and catalysis.

### 3.5 Biomedical Potential of Orange Peel‐Derived AgO‐NPs

The green‐synthesized AgO‐NPs were evaluated for their antibacterial, antioxidant, and antidiabetic potential.

#### 3.5.1 Antimicrobial Activity

**FIGURE 5:**
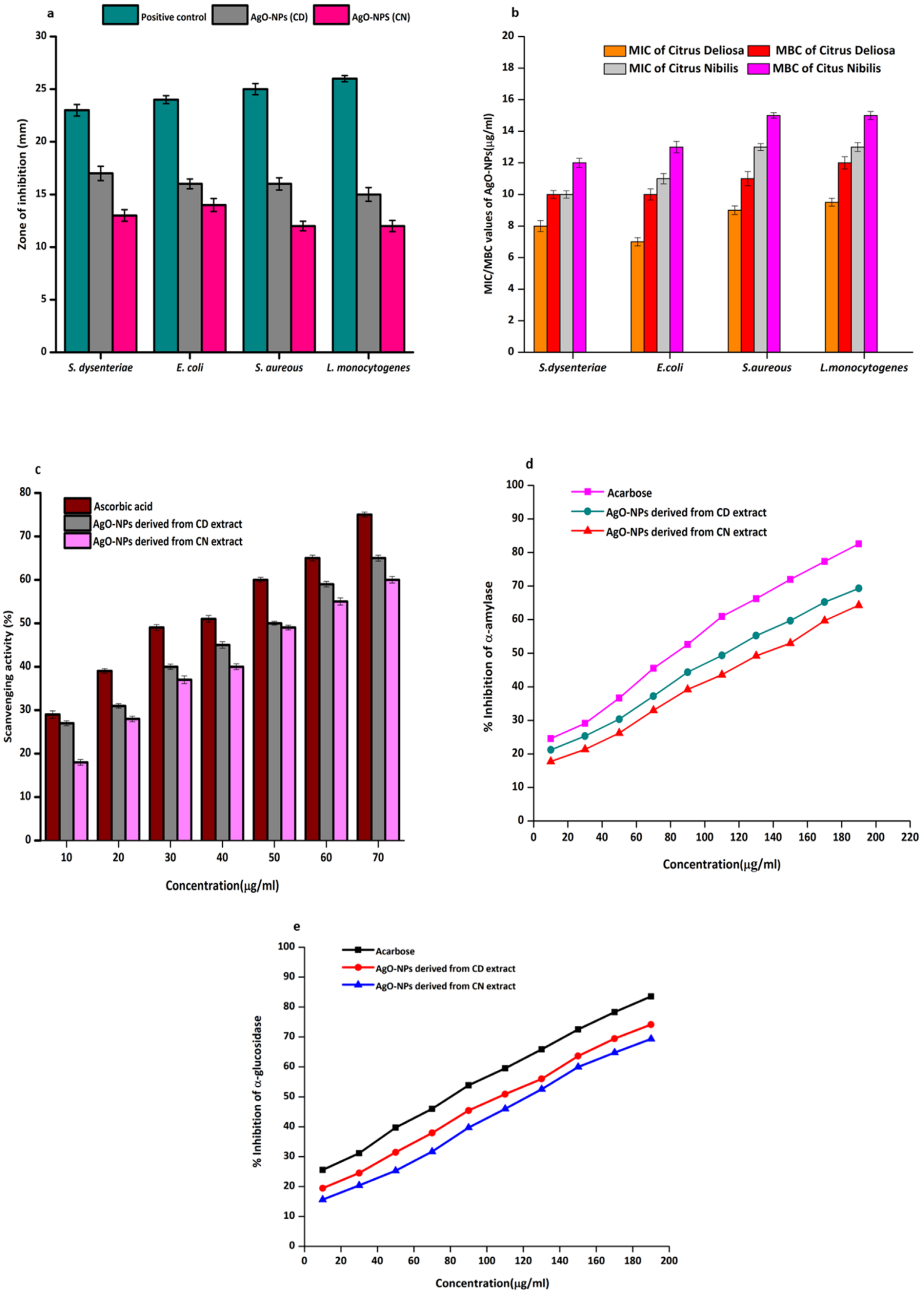
(a) Zone of inhibition, (b) minimum inhibitory concentration and minimum bactericidal concentration values, (c) scavenging activity, and (d, e) antidiabetic potential.

The zone of inhibition (ZOI) was used to measure the antibacterial efficacy of AgO‐NPs derived from CD and CN extract. The antibacterial results are expressed as mean ± SD, and statistical significance was evaluated using one‐way ANOVA followed by Tukey's multiple comparison test (*p* < 0.05) and presented in Figure 5a and Figure S2, clearly showing that the positive control (chloramphenicol) exhibited the highest antibacterial potential across all tested bacterial strains, with a zone of inhibition ranging from approximately 23–26 mm. In comparison, AgO‐NPs derived from the CD extract exhibited a significantly higher zone of inhibition compared to those synthesized from the CN extract. Among the tested pathogens, the ZOI for AgO‐NPs derived from the CD extract ranged from approximately 19–21 mm, while AgO‐NPs derived from the CN extract exhibited zones between 17 and 19 mm. Specifically, AgO‐NPs derived from the CD extract displayed larger inhibition zones of 20.5 ± 0.25 mm for *S. dysenteriae* , 20.9 ± 0.24 mm for *E. coli* , 19.9 ± 0.28 mm for *S. aureus* , and 20.2 ± 0.22 mm for *L. monocytogenes* . Conversely, the AgO‐NPs made from the CN extract exhibited inhibition zones for *S. dysenteriae* (17.5 ± 0.35 mm), *E. coli* (17.9 ± 0.28 mm), *S. aureus* (18.2 ± 0.32 mm), and *L. monocytogenes* (18.6 ± 0.34 mm). This suggests that the antimicrobial properties of AgO‐NPs depend on the method of synthesis, the phytochemistry of the particular species of orange peel material, and the composition of the extract. This difference may have arisen from different levels of some phytochemical compounds, such as polyphenolics, flavonoids, and limonene, which are found in orange peel and act as natural reducing and capping agents during nanoparticle synthesis (Kumar et al. 2020; Sharma 2024a, 2024b). The current results agree with Bhat et al. (2019), Mafhala, Khumalo, et al. (2024), Mafhala, Makhado, et al. (2024), and Trak et al. (2025), who found similar ZOI levels for AgO‐NPs from citrus peel extract.

The antibacterial capability of AgO‐NPs involves disturbing the bacterial cell membrane. AgO‐NPs attach to the cell wall and alter the cell wall structure (Figure 7a), causing increased permeability and loss of the cell contents (Sharma 2024a, 2024b; Guleria, Chawla, et al. 2024; Guleria, Simsek, et al. 2024). In addition, the nanoparticles generate reactive oxygen species (ROS), including superoxide and hydroxyl radicals, which induce oxidative damage to cellular components, including proteins, lipids, and DNA (Daghestani et al. 2020; Kumar 2023). Furthermore, silver ions (Ag^+^) are released from the nanoparticle surface. These ions interact with thiol (‐SH) groups in important bacterial enzymes, disrupting metabolic pathways and inhibiting cellular respiration (Fouda et al. 2023; Singh et al. 2021). Because of their small size and large surface‐to‐volume ratio, AgO‐NPs also penetrate bacterial membranes more effectively. They reach intracellular targets and cause DNA damage or hinder replication (Dabhane et al. 2024; Ramesh et al. 2024).

##### 3.5.1.1 MIC and MBC Values

The minimum inhibitory concentration (MIC) is the lowest amount of an antibacterial agent that can visibly stop the growth of a microorganism. The minimum bactericidal concentration (MBC) is the smallest amount needed to kill 99.9% of bacterial cells. The MIC and MBC were determined using One‐way ANOVA followed by Tukey's multiple comparison test to determine that the results were statistically significant (*p* < 0.05). Figure 5b shows the MIC and MBC values for AgO‐NPs derived from extracts of CD and CN. The data show that AgO‐NPs from CD extract have greater antibacterial effectiveness, as indicated by significantly lower MIC and MBC values compared to those from CN extract. For CN‐derived AgO‐NPs, the MIC values recorded against *S. dysenteriae* , *E. coli* , *S. aureus* , and *L. monocytogenes* were 10, 11, 12, and 13 μg/mL, respectively, while the corresponding MBC values were 12, 13, 15, and 16 μg/mL. In comparison, AgO‐NPs synthesized from CD extract exhibited lower MIC values of 8 μg/mL for *S. dysenteriae* , 9 μg/mL for *E. coli* , 10 μg/mL for *S. aureus* , and 11 μg/mL for *L. monocytogenes* , with the respective MBC values determined as 10, 11, 12, and 13 μg/mL. These results suggest that the CD‐derived nanoparticles have stronger bactericidal and inhibitory effects, likely due to their smaller particle size and higher levels of phytochemicals.

#### 3.5.2 Evaluation of Free Radical Scavenging Activity via the DPPH Method

The free radical scavenging potential of the synthesized AgO‐NPs is illustrated in Figure 5c. The data clearly show a concentration‐dependent increase in antioxidant activity for nanoparticles derived from both CD and CN extracts. As the concentration of nanoparticles increased, a corresponding rise in percentage inhibition was observed. Among the two, AgO‐NPs synthesized from CD extract exhibited the significantly highest antioxidant potential, reaching 65% inhibition at 70 μg/mL. In comparison, CN‐derived AgO‐NPs achieved 60% inhibition at the same concentration. For reference, the standard antioxidant, ascorbic acid, showed a stronger effect with 75% inhibition at 70 μg/mL. These results confirm the dose‐dependent antioxidant behavior of biogenically synthesized nanoparticles as previously reported (Szerlauth et al. 2019; Minhas, Kaleem, et al. 2023; Minhas, Kaur, et al. 2023). The antioxidant activity of AgO‐NPs biogenically derived from citrus peel extract has previously been proposed to result from their ability to neutralize reactive oxygen species (ROS) through multiple complementary mechanisms (Mostafa et al. 2023). These nanoparticles act as free radical scavengers, electron donors, and stabilizing agents. Together, these functions improve their antioxidant effectiveness (Khan et al. 2021). AgO‐NPs made from citrus peel extract show strong antioxidant activity mainly by neutralizing free radicals. This occurs through electron or hydrogen donation, which stabilizes ROS and prevents oxidative damage (Kumar 2023). The bioactive compounds in the extract, such as flavonoids and phenolics, coat the nanoparticles and boost their antioxidant action (Siddiqui et al. 2023; Siddiqui 2023; Sharma 2024a, 2024b). These surface‐bound phytochemicals help the nanoparticles scavenge radicals, inhibit lipid peroxidation, and block metal ion‐induced ROS generation (Yadav 2022a, 2022b; Minhas, Kaleem, et al. 2023; Minhas, Kaur, et al. 2023).

#### 3.5.3 α‐Amylase and α‐Glucosidase Inhibition Assay

The results of the α‐amylase indicate that when the AgO‐NPs concentration is increased, the enzyme inhibition also increases, as illustrated in Figure 5d and Table 2. In the α‐amylase inhibition test, acarbose showed 74.58% inhibition at 190 μg/mL, with an IC₅₀ value of 93.33 μg/mL. Among the nanoparticles tested, those synthesized from CD extract had significantly higher inhibitory activity compared to those from CN. Specifically, CD‐derived AgO‐NPs achieved 69.33% inhibition at 190 μg/mL, with an IC₅₀ value of 110.22 μg/mL. In contrast, CN‐based AgO‐NPs showed 64.22% inhibition at the same concentration, with an IC₅₀ of 121.66 μg/mL. These findings indicate that AgO‐NPs exhibit notable in vitro α‐amylase inhibitory activity, highlighting their potential relevance for further investigation in antidiabetic‐related enzyme studies. However, their effectiveness is slightly lower than that of the standard inhibitor, acarbose. Additionally, α‐glucosidase was also tested, and a similar trend was observed; as the concentration increased, the percentage of inhibition also increased (Figure 5e and Table 3). In the α‐glucosidase inhibition test, acarbose had an inhibitory effect of 78.57% at 190 μg/mL, with an IC₅₀ value of 95.08 μg/mL. AgO‐NPs made from CD peel extract showed 73.17% inhibition at the same concentration, with an IC₅₀ of 114.62 μg/mL. Meanwhile, CN‐derived AgO‐NPs exhibited a notable inhibitory effect of 68.39% at 190 μg/mL, with an IC₅₀ value of 125.56 μg/mL. The results obtained are similar to those from previous studies by Chen et al. (2022), Ibrahim (2023), Trak (2025) and Trak et al. (2025). They also noted the antidiabetic potential of AgO‐NPs synthesized from citrus peel extract.

**TABLE 2:** α‐Amylase inhibitory effects of acarbose and AgO‐NPs derived from CD and CN peel extracts.

Concentration (μg/mL)	%Inhibition of α‐amylase					
	Acarbose	IC_50_ value (μg/mL)	AgO‐NPs derived from *Citrus deliosa*	IC_50_ value (μg/mL)	AgO‐NPs derived from *Citrus nobilis*	IC_50_ value (μg/mL)
10	24.58 ± 0.35	93.33	21.21 ± 0.24	110.22	19.75 ± 0.26	121.66
30	29.12 ± 0.55		25.32 ± 0.55		21.37 ± 0.35	
50	35.66 ± 0.22		32.25 ± 0.48		29.23 ± 0.15	
70	43.52 ± 0.48		38.23 ± 0.37		32.96 ± 0.48	
90	49.59 ± 0.54		44.32 ± 0.47		39.22 ± 0.53	
110	54.95 ± 0.48		50.99 ± 0.27		47.60 ± 0.38	
130	59.23 ± 0.48		55.24 ± 0.25		51.22 ± 0.29	
150	64.98 ± 0.14		59.68 ± 0.35		54.99 ± 0.38	
170	69.35 ± 0.57		63.24 ± 0.48		60.69 ± 0.28	
190	74.58 ± 0.35		69.33 ± 0.29		64.26 ± 0.30	

**TABLE 3:** α‐Glucosidase inhibitory effects of acarbose and AgO‐NPs derived from CD and CN peel extracts.

Concentration (μg/mL)	%Inhibition of α‐glucosidase					
	Acarbose	IC_50_ value (μg/mL)	AgO‐NPs derived from *Citrus deliosa*	IC_50_ value (μg/mL)	AgO‐NPs derived from *Citrus nobilis*	IC_50_ value (μg/mL)
10	26.57 ± 0.33	95.08	23.43 ± 0.33	114.62	20.62 ± 0.26	125.56
30	29.11 ± 0.52		28.51 ± 0.56		24.35 ± 0.33	
50	35.72 ± 0.22		33.43 ± 0.53		29.30 ± 0.15	
70	42.97 ± 0.25		38.92 ± 0.45		35.65 ± 0.47	
90	48.87 ± 0.48		44.43 ± 0.55		41.71 ± 0.54	
110	55.56 ± 0.54		49.02 ± 0.33		46.99 ± 0.38	
130	62.88 ± 0.25		56.99 ± 0.53		52.50 ± 0.27	
150	68.52 ± 0.59		62.65 ± 0.34		58.98 ± 0.37	
170	73.35 ± 0.57		69.45 ± 0.48		62.78 ± 0.23	
190	78.57 ± 0.13		73.17 ± 0.35		68.39 ± 0.30	

There are multiple pathways through which AgO‐NPs show antidiabetic potential (Figure 7b). One of the main mechanisms involves inhibiting digestive enzymes like α‐amylase and α‐glucosidase. These enzymes break down polysaccharides into glucose by reducing the activity of these enzymes (Jini et al. 2022). AgO‐NPs help manage postprandial blood sugar levels, similar to standard antidiabetic drugs like acarbose (Mafhala, Khumalo, et al. 2024; Mafhala, Makhado, et al. 2024; Trak et al. 2025; Trak 2025). Furthermore, phytochemicals from citrus peel work together with the nanoparticles. This enhances enzyme inhibition and facilitates the regulation of metabolism (Chen et al. 2022).

### 3.6 Environmental Potential of Orange Peel‐Derived AgO‐NPs

**FIGURE 6:**
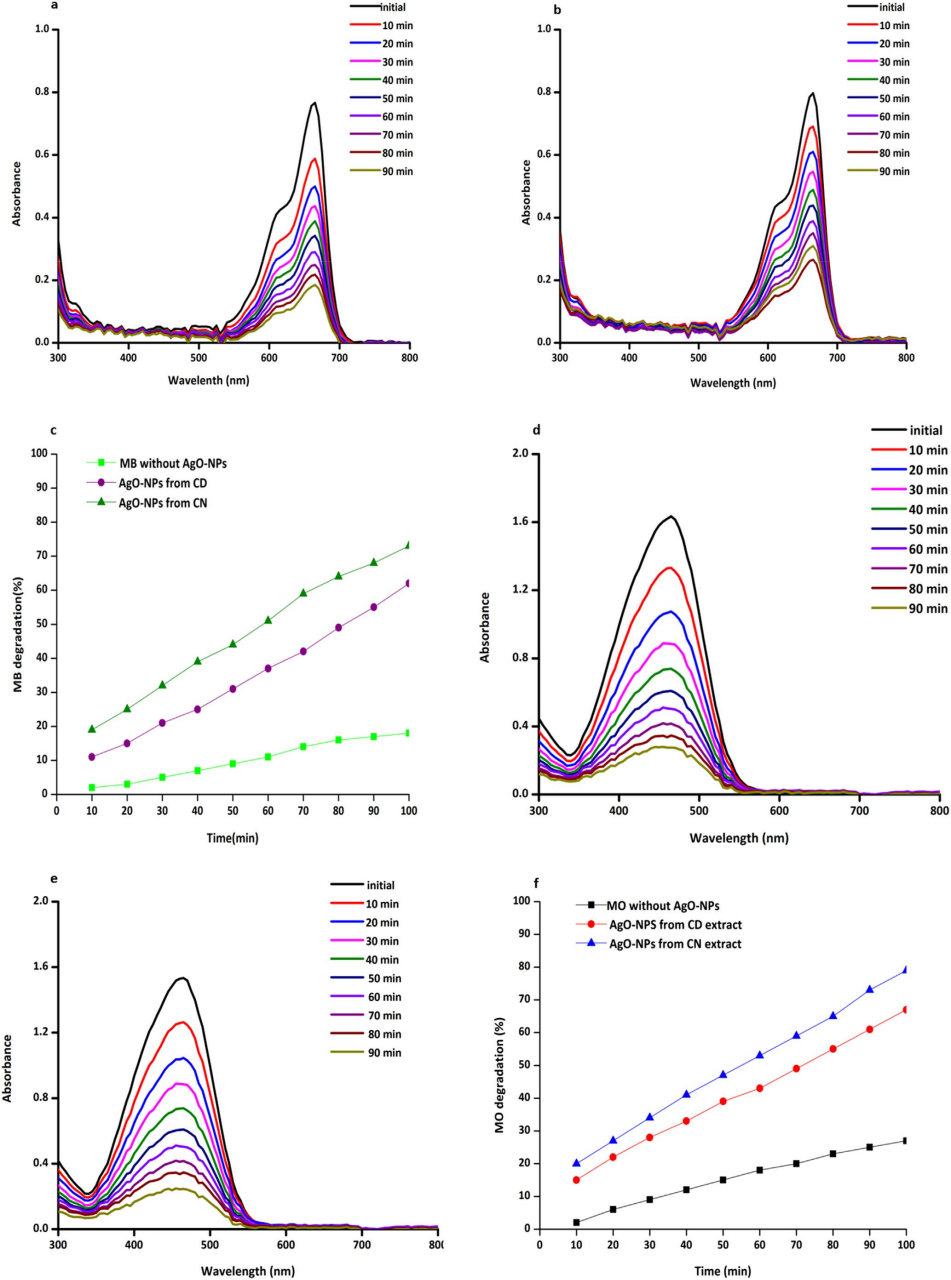
Methylene blue degradation by AgO‐NPs derived from (a) CD, (b) CN peel extract, (c) % degradation of MB, (d) methyl orange degradation by AgO‐NPs derived from (e) CD, and (f) CN peel extract.

**FIGURE 7:**
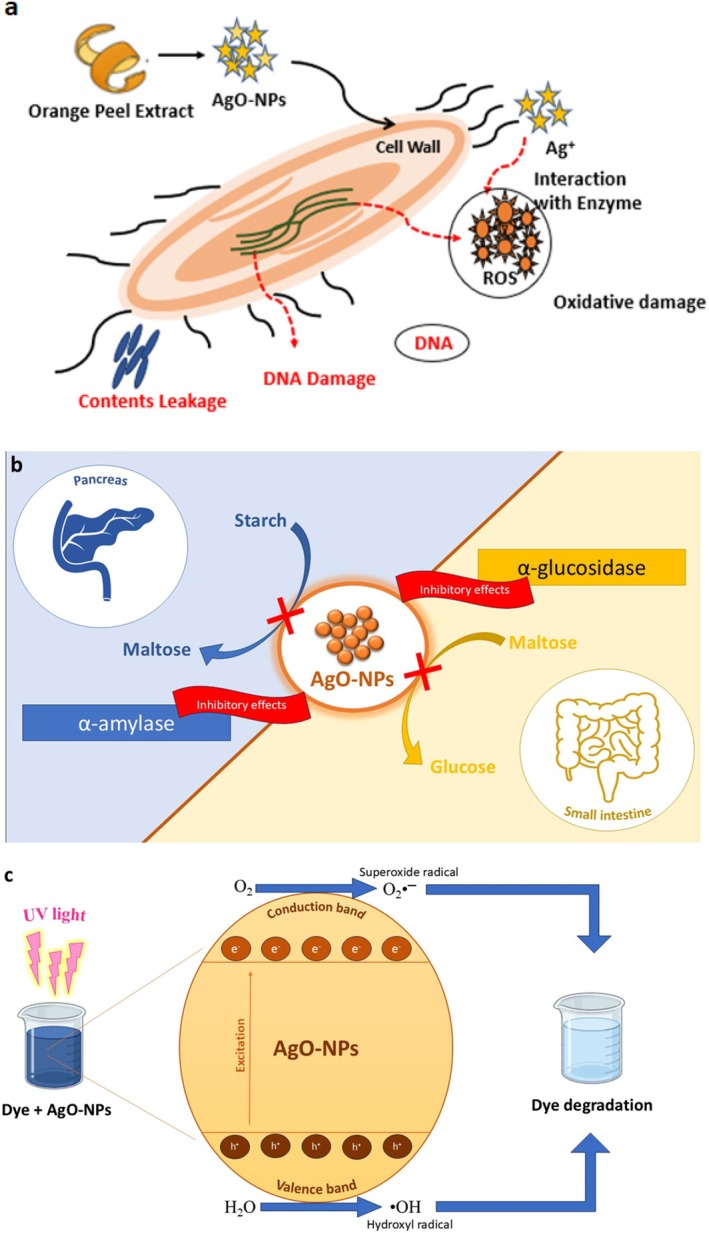
(a) Antibacterial (b) Antidiabetic (c) Dye degradation mechanisms of AgO‐NPs derived from citrus peel extract.

The photocatalytic performance of AgO‐NPs synthesized by using CD and CN peel extracts was evaluated for degrading methylene blue (MB) and methyl orange (MO) under ultraviolet (UV) light irradiation. The degradation process is measured by measuring the spectral absorbance of each dye at regular intervals, as shown in Figure 6a–f and Table 4. The data clearly show that AgO‐NPs made with CD extract have better photocatalytic activity compared to those made with CN extract. After 90 min of UV exposure, the degradation efficiency of MB reached 73.51% with CD‐derived AgO‐NPs, while CN‐derived nanoparticles achieved a lower efficiency of 69.94% (Figure 6a–c). For MO, CD‐based nanoparticles showed 71.68% degradation, whereas CN‐based AgO‐NPs reached 65.98% under the same conditions (Figure 6d–f). These results suggest that the higher photocatalytic efficiency of CD‐derived AgO‐NPs may be due to differences in nanoparticle size, surface properties, or phytochemical composition of the plant extracts. Overall, the findings highlight the greater effectiveness of CD‐derived AgO‐NPs in breaking down organic dye pollutants, making them more suitable for photocatalytic wastewater treatment. Light generates photons that excite electrons from the valence band to the conduction band in AgO‐NPs and generates electron–hole pairs (Figure 7c). This process begins with electron–hole pair production and leads to redox reactions at the surface of the nanoparticles (de Oliveira Guidolin et al. 2021). The electrons (e^−^) reduce molecular oxygen (O_2_) to molecular superoxide (•O_2_^−^), while the holes (h^+^) oxidize water (H_2_O) or hydroxide (·OH) ions to create hydroxyl radicals (•OH) (Al‐Radadi et al. 2022). Both reactive species are extremely oxidative and provide a means to decompose complex dye molecules to products that are less harmful (i.e., CO_2_ and H_2_O) (Kumar 2023; Sharma 2024a, 2024b). The phytochemicals in the extract of citrus peels, which include flavonoids, phenolics, and limonene, also contribute to the stabilization of the nanoparticles and improve charge separation and electron transfer, therefore improving the photocatalytic performances of the nanoparticles (Siddiqui et al. 2023; Siddiqui 2023). AgO‐NPs derived from citrus peels can be used as a viable and environmentally friendly photocatalyst for degrading synthetic dyes in wastewater treatment.

**TABLE 4:** UV photodegradation kinetics of methylene blue and methyl orange by AgO‐NPs derived from CD and CN peel extracts.

Photocatalytic activity		AgO‐NPs derived from CD peel extract			AgO‐NPs derived from CN peel extract		
		Degradation (%)	Rate constant (min^−1^)	Regression coefficient (*R* ^2^)	Degradation (%)	Rate constant (min^−1^)	Regression coefficient (*R* ^2^)
Under UV irradiation	Methylene blue	73.51	0.0148	0.992	69.94	0.0134	0.982
	Methyl orange	72.68	0.0144	0.987	65.98	0.0120	0.978

## 4 Conclusion

The current study demostrates a safe and cost‐effective way to synthesize AgO‐NPs using peel extracts from CD and CN. The phytochemical components of both extracts played a crucial role in reducing and stabilizing the nanoparticles. To find the best conditions for stabilizing the nanoparticles, the extract concentrations were varied from 0.5% to 2.5% for CD and 1% to 5% for CN. The localized surface plasmon resonance (LSPR) absorption peaks were observed between 420 and 460 nm with UV–Visible spectroscopy. Additionally, Dynamic Light Scattering (DLS) analysis showed that the synthesized nanoparticles had hydrodynamic diameters ranging from 14 to 55 nm. Further, Fourier Transform Infrared Spectroscopy was used to identify the surface functional groups. Moreover, other characterization methods, including Scanning Electron Microscopy (with Energy Dispersive X‐ray Spectroscopy, Thermal Gravimetric Analysis, and X‐ray Diffraction), confirmed the successful synthesis of stable, crystalline, and thermally robust AgO‐NPs. In comparative analysis, AgO‐NPs from CD showed better biomedical potential. In antimicrobial tests, CD‐based nanoparticles had strong activity, with inhibition zones between 19 and 21 mm against *Staphylococcus aureus* , *Streptococcus lactis* , *Escherichia coli* , and *Salmonella typhimurium* . These nanoparticles also showed significant antioxidant capacity, achieving 69.33% DPPH radical scavenging at a concentration of 190 μg/mL. Moreover, antidiabetic tests revealed strong effects on digestive enzymes, with 69.33% inhibition of α‐amylase and 73.17% inhibition of α‐glucosidase at the same concentration. Apart from their biomedical uses, the CD‐derived AgO‐NPs demonstrated impressive photocatalytic performance. Under ultraviolet light, they effectively broke down MB (73.65%) and MO (71.68%) in 90 min. These multifunctional properties reveal the potential of CD‐based AgO‐NPs as valuable agents for both medical and environmental applications. Overall, this study highlights the possibility of using agricultural waste (orange peel) in the green synthesis of silver oxide nanoparticles and their wide range of applications in medicine and environmental clean‐up.

## Author Contributions

**Prabhleen Kaur:** methodology, writing – review and editing. **Diksha Sharma:** methodology, data curation, writing – original draft. **Samriti Guleria:** conceptualization, methodology, investigation, resources, writing – original draft. **Prashant Anil Pawase:** writing – review and editing. **Aparajita Bhasin:** data curation, writing – review and editing. **Halis Simsek:** conceptualization, data curation, writing – review and editing.

## Funding

The financial assistance was provided by the Department of Science and Technology (DST), Government of India, under the FIST program (Sanction No. SR/FST/COLLEGE‐/2019/814, dated 07.01.2020).

## Conflicts of Interest

The authors declare no conflicts of interest.

## Supporting information


**Appendix S1:** The supplementary material supports the green synthesis, characterization, and antibacterial activity of silver oxide nanoparticles (AgO‐NPs) derived from citrus peel extracts. Figure S1 presents the proposed synthesis mechanism, highlighting the role of phytochemicals as reducing and stabilizing agents, while Figure S2 demonstrates their antibacterial efficacy. FT‐IR results (Tables S1 and S2) confirm the presence of functional groups associated with biomolecules involved in nanoparticle formation, and the Ag–O band (~695 cm^−1^) verifies successful synthesis.
**Figure S1:** Proposed Mechanism of AgO‐NPs derived from Citrus peel extract.
**Figure S2:** Antibacterial potential of AgO‐NPs derived from CD and CN.
**Table S1:** FT‐IR absorption bands and corresponding functional groups present in CD peel extract and the derived AgO‐NPs.
**Table S2:** FT‐IR absorption bands and corresponding functional groups present in CN peel extract and the derived AgO‐NPs.

## Data Availability

The data that support the findings of this study are available on request from the corresponding author. The data are not publicly available due to privacy or ethical restrictions.
