# Supplementary material for: Exploring the Biomedical and Environmental Application of Silver Oxide Nanoparticles Derived From Citrus sinensis Peel: A Valorization Approach
Source: Food Sci Nutr. 2026 May 19;14(5):e71818. doi: 10.1002/fsn3.71818 (PMC13184997; doi:10.1002/fsn3.71818)
Supplement: Supplementary file 1 — Appendix S1: The supplementary material supports the green synthesis, characterization, and antibacterial activity of silver oxide nanoparticles (AgO‐NPs) derived from citrus peel extracts. Figure S1 presents the proposed synthesis mechanism, highlighting the role of phytochemicals as reducing and stabilizing agents, while Figure S2 demonstrates their antibacterial efficacy. FT‐IR results (Tables S1 and S2) confirm the presence of functional groups associated with biomolecules involved in nanoparticle formation, and the Ag–O band (~695 cm−1) verifies successful synthesis. Figure S1: Proposed Mechanism of AgO‐NPs derived from Citrus peel extract. Figure S2: Antibacterial potential of AgO‐NPs derived from CD and CN. Table S1: FT‐IR absorption bands and corresponding functional groups present in CD peel extract and the derived AgO‐NPs. Table S2: FT‐IR absorption bands and corresponding functional groups present in CN peel extract and the derived AgO‐NPs. [file FSN3-14-e71818-s001.docx]

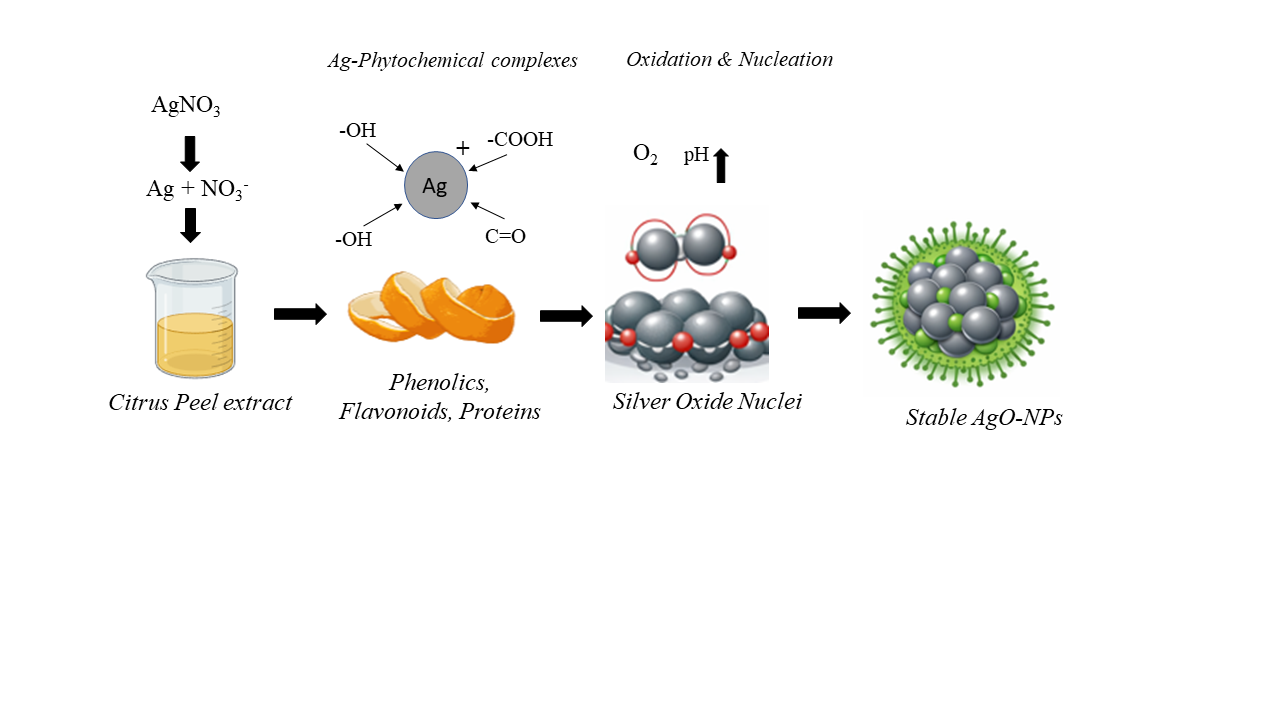


**Figure 1S:** Proposed Mechanism of AgO-NPs derived from Citrus peel extract

| 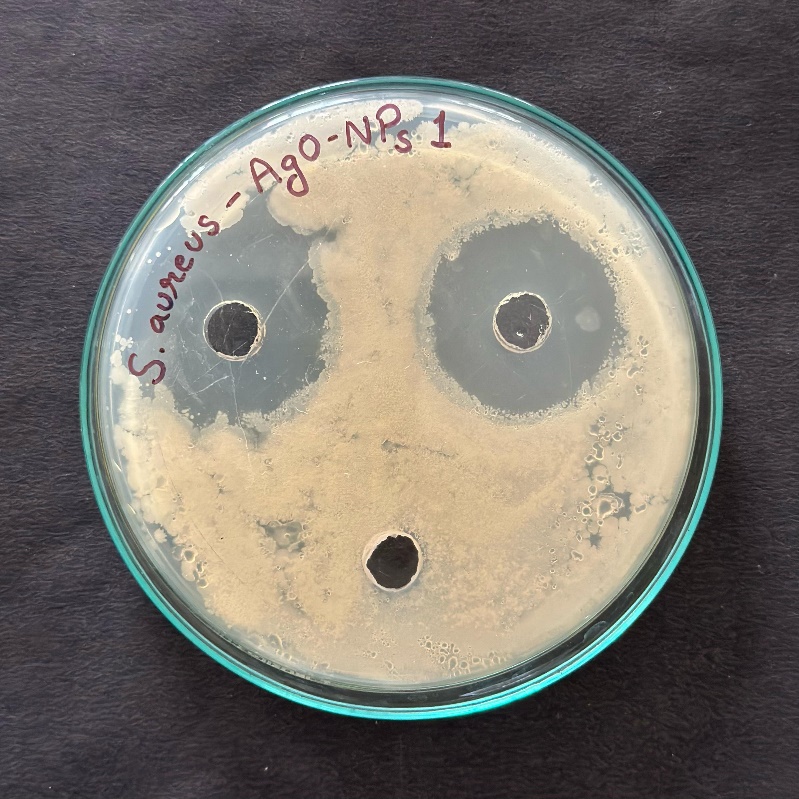 | 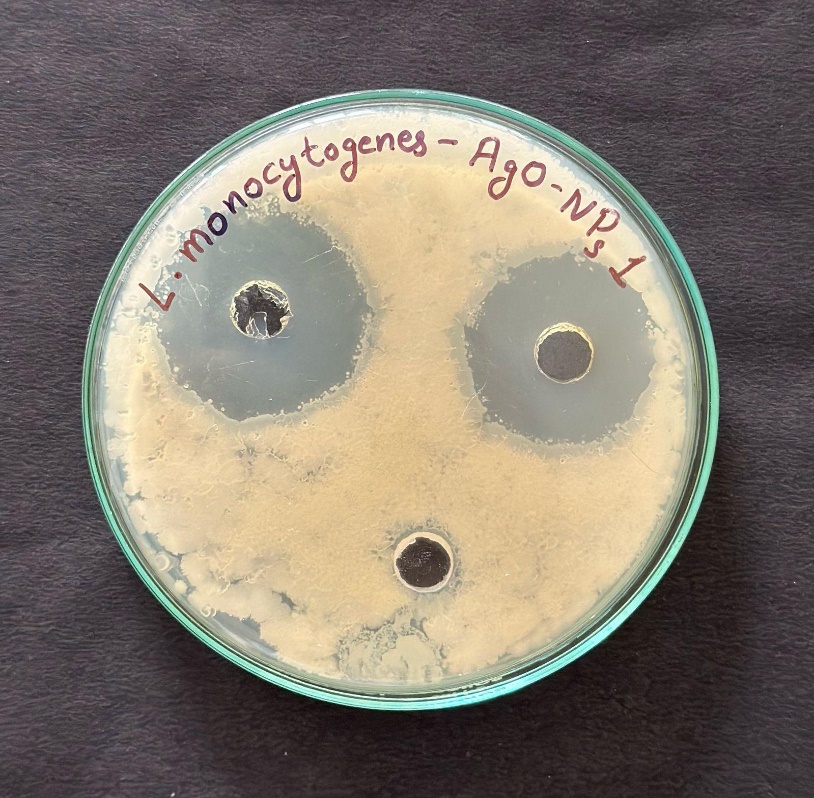 |
| --- | --- |
| 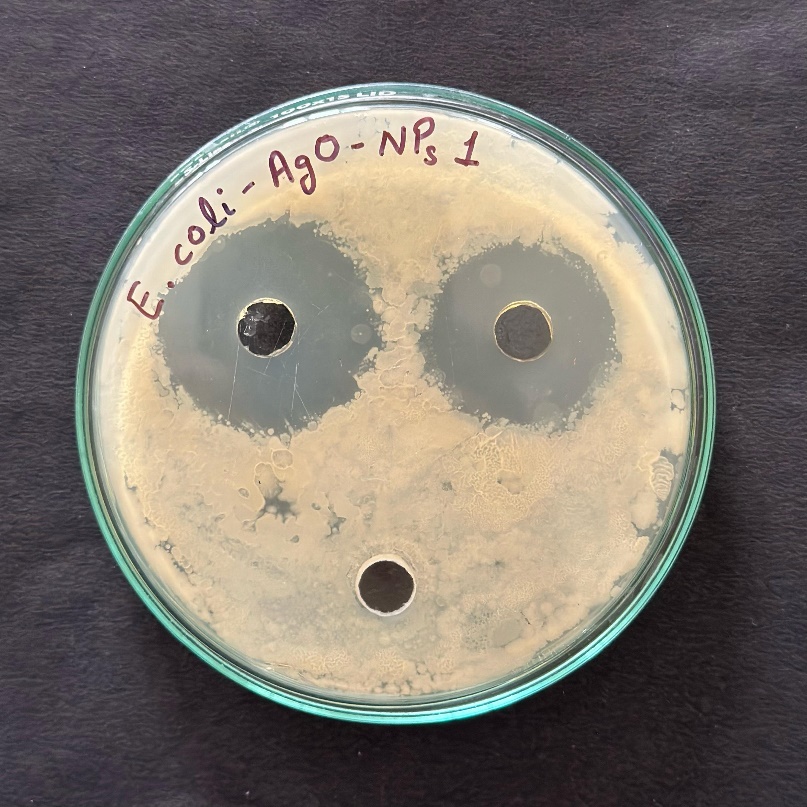 | 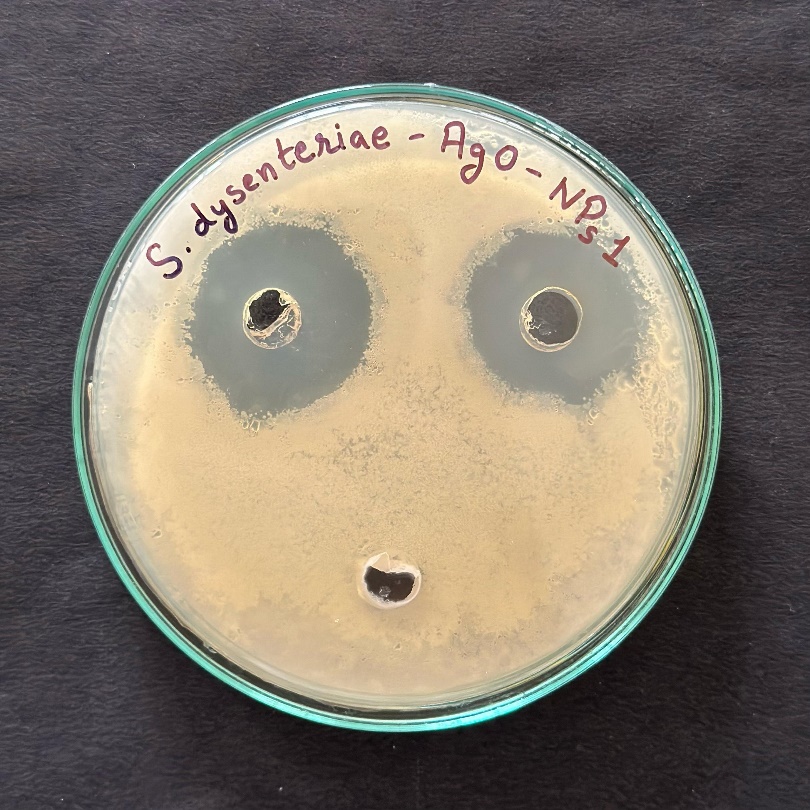 |
| 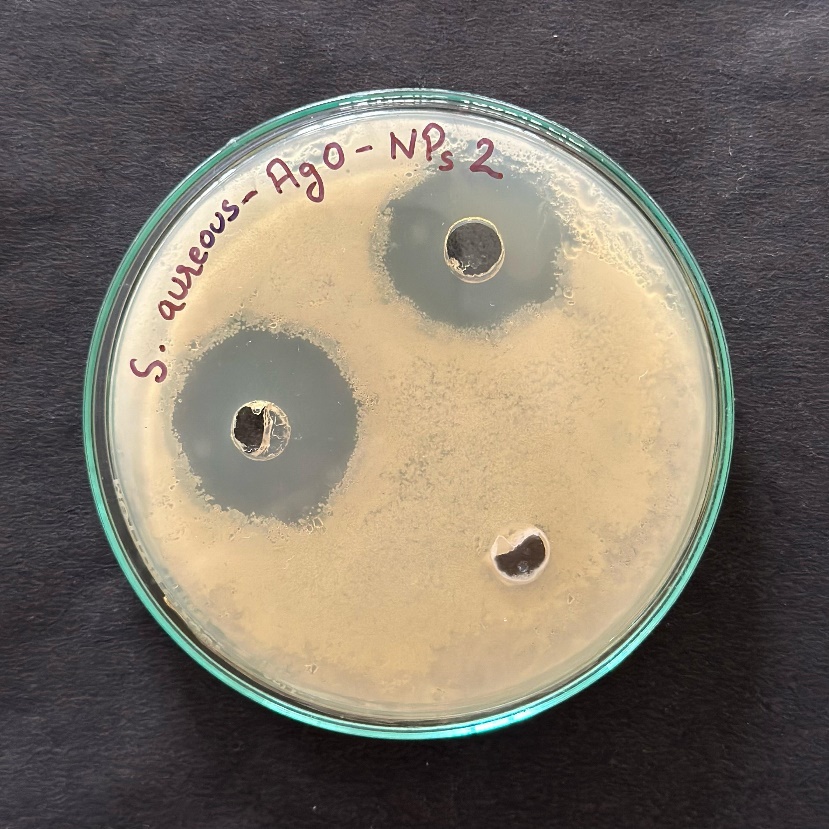 | 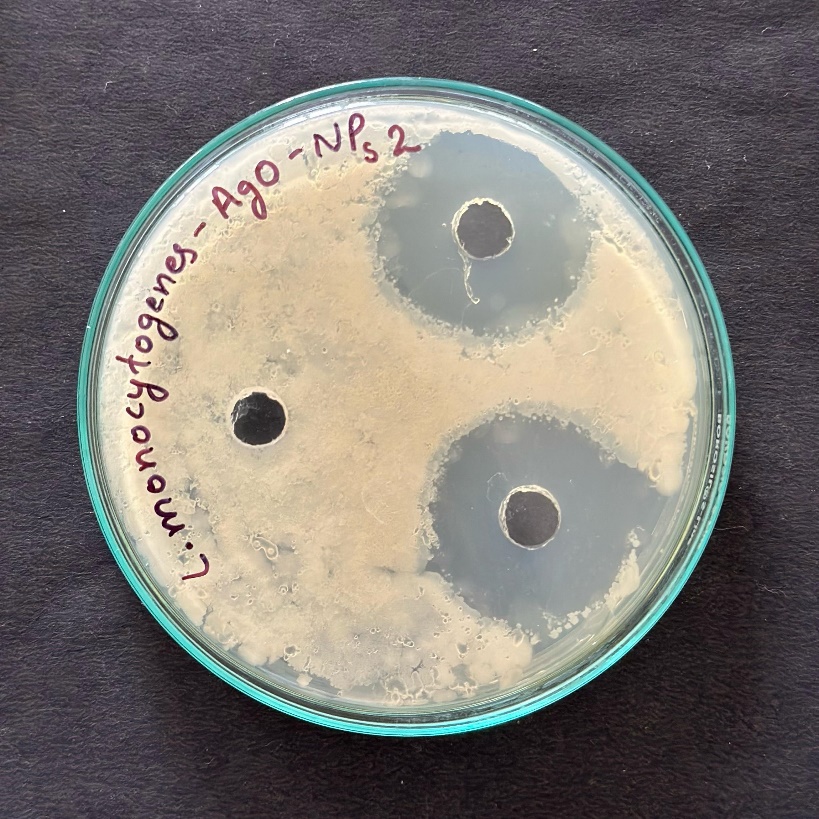 |
| 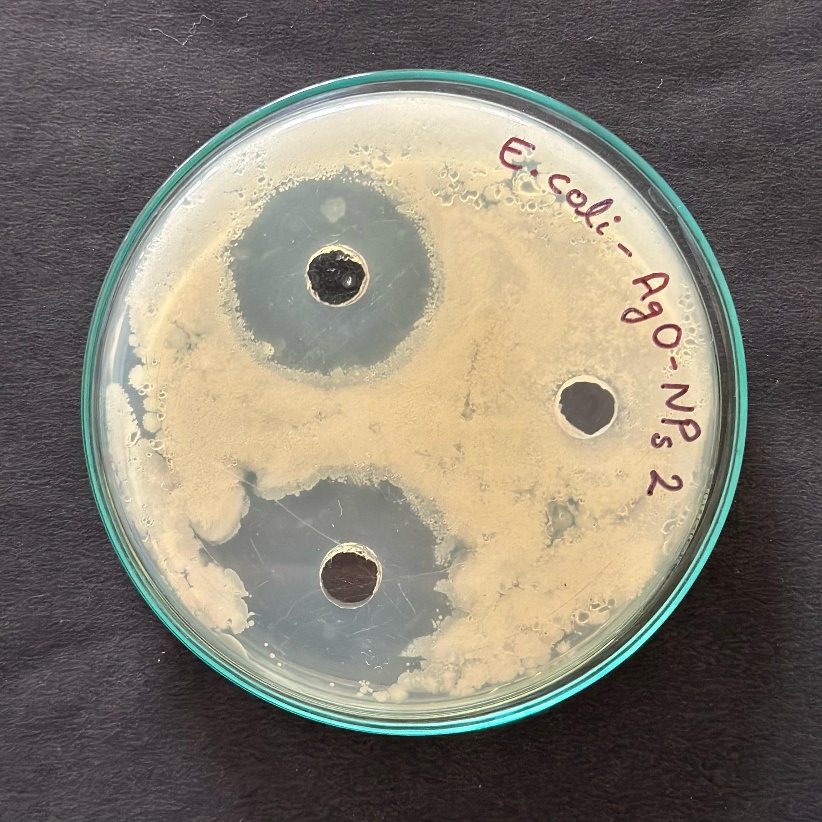 | 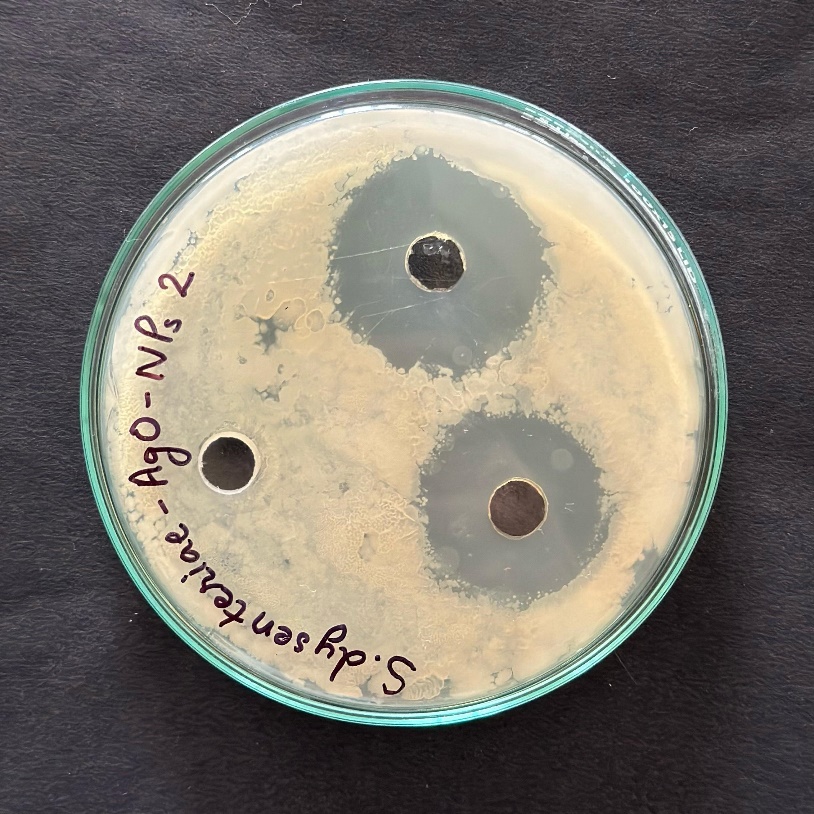 |

**Figure 2S:** Antibacterial potential of AgO-NPs derived from CD and CN

**Table 1S:** FT-IR absorption bands and corresponding functional groups present in CD peel extract and the derived AgO-NPs.

| **Wave number (cm⁻¹)** | **CD peel extract** | **AgO-NPs** | **Probable functional group** | **Biomolecule Class** |
| --- | --- | --- | --- | --- |
| 3360–3390 | 3365 | 3368 | O–H stretching | Phenols, alcohols |
| 2920–2930 | 2922 | 2925 | C–H stretching | Lipids |
| 1630–1650 | 1642 | 1644 | C=O / Amide I | Proteins, polyphenols |
| 1440–1455 | 1450 | 1452 | C–H bending / COO⁻ | Carboxylic acids |
| 1240–1260 | 1242 | 1245 | C–O stretching | Phenolic compounds |
| 1060–1080 | 1065 | 1068 | C–O–C stretching | Carbohydrates |
| 680–720 |  | 695 | Ag–O vibration | Silver oxide |

**Table 2S:** FT-IR absorption bands and corresponding functional groups present in CN peel extract and the derived AgO-NPs.

| **Wave number (cm⁻¹)** | **CN peel extract** | **AgO-NPs** | **Probable functional group** | **Biomolecule Class** |
| --- | --- | --- | --- | --- |
| 3360–3390 | 3375 | 3378 | O–H stretching | Phenols, alcohols |
| 2920–2930 | 2924 | 2923 | C–H stretching | Lipids |
| 1630–1650 | 1639 | 1645 | C=O / Amide I | Proteins, polyphenols |
| 1440–1455 | 1449 | 1452 | C–H ending / COO⁻ | Carboxylic acids |
| 1240–1260 | 1245 | 1247 | C–O stretching | Phenolic compounds |
| 1060–1080 | 1075 | 1066 | C–O–C stretching | Carbohydrates |
| 680–720 |  | 695 | Ag–O vibration | Silver oxide |
